# The role of organizational context in moderating the effect of research use on pain outcomes in hospitalized children: a cross sectional study

**DOI:** 10.1186/s12913-017-2029-2

**Published:** 2017-01-23

**Authors:** Janet Yamada, Janet E. Squires, Carole A. Estabrooks, Charles Victor, Bonnie Stevens, CIHR Team in Children’s Pain, Fiona Campbell, Christine Chambers, Janice Cohen, Greta Cummings, G Allen Finley, Denise Harrison, Liisa Holsti, Céleste Johnston, Margot Latimer, Shoo Lee, Sylvie LeMay, Patrick McGrath, Judy Rashotte, Christina Rosmus, Doris Sawatzky-Dickson, Shannon Scott, Souraya Sidani, Jennifer Stinson, Anna Taddio, Fay Warnock, Andrew R. Willan

**Affiliations:** 1 Daphne Cockwell School of Nursing, Ryerson University, Ottawa, ON Canada; 2 Faculty of Health Sciences, School of Nursing, University of Ottawa, Ottawa, ON Canada; 3 Clinical Epidemiology Program, Ottawa Hospital Research Institute, Ottawa, ON Canada; 4 Faculty of Nursing, University of Alberta, Edmonton, AB Canada; 5 University of Toronto, Ottawa, ON Canada; 6 Lawrence S Bloomberg, Faculty of Nursing, University of Toronto, Ottawa, ON Canada; 7 Child Health Evaluation Sciences, Research Institute, The Hospital for Sick Children, 686 Bay Street, Room 06.9712, Ottawa, ON M5G 1X8 Canada

**Keywords:** organizational context, work environment, culture, leadership, evaluation, knowledge translation, pain, children

## Abstract

**Background:**

Despite substantial research on pediatric pain assessment and management, health care professionals do not adequately incorporate this knowledge into clinical practice. Organizational context (work environment) is a significant factor in influencing outcomes; however, the nature of the mechanisms are relatively unknown. The objective of this study was to assess how organizational context moderates the effect of research use and pain outcomes in hospitalized children.

**Methods:**

A cross-sectional survey was undertaken with 779 nurses in 32 patient care units in 8 Canadian pediatric hospitals, following implementation of a multifaceted knowledge translation intervention, Evidence-based Practice for Improving Quality (EPIQ). The influence of organizational context was assessed in relation to pain process (assessment and management) and clinical (pain intensity) outcomes. Organizational context was measured using the Alberta Context Tool that includes: leadership, culture, evaluation, social capital, informal interactions, formal interactions, structural and electronic resources, and organizational slack (staff, space, and time). Marginal modeling estimated the effects of instrumental research use (direct use of research knowledge) and conceptual research use (indirect use of research knowledge) on pain outcomes while examining the effects of context.

**Results:**

Six of the 10 organizational context factors (culture, social capital, informal interactions, resources, and organizational slack [space and time]) significantly moderated the effect of instrumental research use on pain assessment; four factors (culture, social capital, resources and organizational slack time) moderated the effect of conceptual research use and pain assessment. Only two factors (evaluation and formal interactions) moderated the effect of instrumental research use on pain management. All organizational factors except slack space significantly moderated the effect of instrumental research use on pain intensity; informal interactions and organizational slack space moderated the effect of conceptual research use and pain intensity.

**Conclusions:**

Many aspects of organizational context consistently moderated the effects of instrumental research use on pain assessment and pain intensity, while only a few influenced conceptual use of research on pain outcomes. Organizational context factors did not generally influence the effect of research use on pain management. Further research is required to further explore the relationships between organizational context and pain management outcomes.

## Background

Hospitalized children experience frequent painful procedures [1]. Despite a growing body of research on effective pain assessment and management strategies, health care professionals are not consistently using this evidence to achieve best practice [2]. For example, less than 30% of children have pain assessed with a validated pain measure or have pain relieving interventions accompanying acute painful procedures [1, 2]. Our previous research in 32 patient care units in eight Canadian pediatric hospitals determined that a multifaceted knowledge translation (KT) intervention, Evidence-based Practice for Improving Quality (EPIQ) [3, 4] improved pain assessment and management practices of healthcare professionals, and decreased pain intensity in hospitalized children compared to standard care [5]. However, there was substantial variation in outcomes across hospital units. To address this variability, our current study aimed to determine the influence of organizational context in moderating the effect of research use and pain outcomes.

Nurses play a major role in pain assessment and management of hospitalized children. Expanding their knowledge on how to assess pain using validated pain measures and manage pain using evidence-based interventions provides a comprehensive basis for practice. However, implementing research evidence in the practice setting is complex and is influenced by individual (e.g. knowledge, attitudes and beliefs) and organizational contextual (i.e. work environment factors such as leadership, interactions, resources) factors. Most investigations have traditionally focused on individual provider factors and behaviours in decision making to enhance patient outcomes [6]. While individual factors have been closely associated with nurses’ success in implementing research into practice, many barriers exist [7]. There is now growing evidence that organizational context may have a greater impact on successful implementation of research evidence compared to individual healthcare professional factors [8, 9]. In the Promoting Action on Research in Health Services (PARiHS) framework [10–12], Kitson proposed that successful implementation of research in an organization is a function of the interplay of context, evidence, and facilitation; where a “high context” with collaborative cultures, strong leadership, and appropriate monitoring and feedback systems being receptive to change [12].

Several studies illustrate the influence of both individual nurse factors and organizational context factors on predicting nurses’ use of research. Squires et al. [13] identified organizational context as a significant *predictor* of pediatric nurses’ research use, using the Alberta Context Tool (ACT) [14]. The proportion of nurses possessing a baccalaureate degree or higher and unit culture significantly predicted nurses’ instrumental research use (IRU) (i.e., direct use of research; when evidence is translated to a format such as a guideline or protocol and used for making decisions about patient care) while leadership, culture, evaluation (feedback of patient data to the unit), formal interactions, informal interactions, organizational slack [availability of resources (i.e., space, staff, time) which allow the unit to adapt successfully to internal or external pressures for change] (space), and unit specialty predicted conceptual research use (CRU) (i.e., indirect use of research; that enlightens or informs the user’s attitudes or beliefs about research, but may not result in clinical practice changes [13, 14]). Estabrooks et al. [15] reported that social capital, organizational slack (staffing and time), the number of informal interactions and unit type were significant predictors of IRU by care aides to residents in Canadian nursing homes. Significant predictors of CRU included evaluation, structural resources and organizational slack (time).

Little is known about how organizational context influences pain processes and clinical outcomes. The objective of this study was to assess how organizational context moderates the effect of nurses research use (IRU and CRU) on pain outcomes in hospitalized children. We hypothesized that organizational context would positively influence the relationship between research use and pain process and clinical outcomes.

## Methods

### Design, sample and setting

#### Design

Our research team, the Canadian Institutes of Health Research (CIHR) Team in Children’s Pain research conducted two studies between 2008-2013. These studies included the: (a) EPIQ Intervention Study - a prospective cohort design with repeated measures – to determine the effectiveness of a multifaceted KT intervention, EPIQ, on nurses’ pain practices and clinical pain outcomes [5]; and (b) Context Study (reported in this paper) - a cross-sectional survey design - to determine how organizational context influenced the relationship between nurses’ research use and pain practice and clinical outcomes.

#### Sample and setting

Pediatric tertiary level hospitals in Canada who had four or more distinct units with 15 or more beds were eligible to participate in the EPIQ and Context studies. Hospital units were included if: (a) they were separately located within the hospital; and (b) patients were exposed to acute procedural pain, where (c) pharmacological, physical, and psychological interventions were available. Psychiatric units and emergency units were excluded as patients were frequently not hospitalized for the requisite 24-h data collection period. Eight hospitals met the criteria and agreed to participate. At each hospital site, four units were included, with at least one medical, one surgical, and one critical care unit [4, 5]. Nurses on participating units were eligible for the Context study if they had worked on the unit for a minimum of six months in a full-time, part-time, or casual capacity; and spoke and read English or French. Trainees were excluded.

The EPIQ study sample comprised 964 children (i.e., approximately 30 from each of the 32 hospital units) whose medical records were reviewed to record pain assessment and management outcomes. Pain intensity data were prospectively collected on an additional 640 children (i.e., 20 children on each of the 32 hospital units) during a scheduled painful procedure [4, 5].

The Context study sample included 779 (of 2157 eligible) nurses across participating hospital units who completed a staff survey (36% response rate) (Table 1). Data from individual nurses and patients were aggregated to the unit level. Research ethics approvals were obtained in accordance with individual institutional Research Ethics Boards at all study sites.

**Table 1:** Characteristics of Nurse Respondents (*N* = 779)

Characteristic	N (%)
Primary role	
- RN	733 (94.1)
- LPN	19 (2.4)
- RPN	9 (1.2)
- Other (identified as nurse)	18 (2.3)
Age	
- 20 to 29	269 (34.5)
- 30 to 39	211 (27.1)
- 40 to 49	155 (19.9)
- 50 to 59	127 (16.3)
- 60+	16 (2.1)
- not reported	1 (0.1)
Sex	
- Male	44 (5.7)
- Female	734 (94.2)
- not reported	1 (0.1)
Education	
- Diploma/Certificate	244 (31.3)
- Bachelors	507 (65.1)
- MD	2 (0.3)
- Masters	23 (3.0)
- PhD	0 (0.0)
- not reported	3 (0.4)
Employment status	
- FT	474 (60.9)
- PT	273 (35.0)
- Casual	31 (4.0)
- not reported	1 (0.1)
Specialized course	
- Yes	235 (30.2)
- No	543 (69.7)
- not reported	1 (0.1)

### Procedure

#### Intervention

As the EPIQ intervention and study results are reported elsewhwere [4, 5], we describe them here briefly only to clarify how the EPIQ intervention is related to the current Context study. The EPIQ intervention was implemented over a 15-month period in 16 units across eight hospital sites (two units per site) and compared to standard care in 16 units (two units per site) in the same hospitals. EPIQ consists of a: (1) *preparation phase* where units establish a team of implementation leads (e.g. clinical nurse specialist/ practitioner, nurse educator), examine the unit’s baseline pain assessment and management practices, review published research evidence, and determine their pain practice change aim(s); and (2) *implementation phase*, where the implementation leads develop, implement, and evaluate evidence-based KT strategies (e.g. educational sessions, reminders, audit and feedback) in four, three-month cycles of change, and monitor their progress [4, 5]. Organizational context data were collected from staff in each unit participating in the EPIQ study.

### Study variables and measures

Data were collected to determine the influence of organizational context on the relationship between research use and pain outcomes.

#### Organizational context variables

Organizational context was measured using the Alberta Context Tool (ACT) [14], which was part of the staff survey. The ACT assesses health care professionals’ perceptions of modifiable aspects of the work environment. Developed for acute care (adult) hospitals [14], the ACT was successfully adapted for use in pediatric hospitals [16] and was translated into French. All eligible nurses on the participating hospital units completed the ACT Pediatric Nurse Version online. It contained 56 items representing the 10 context concepts. Higher scores represent a more “favorable” context. The leadership, culture and evaluation concepts align with context as conceptualized in the PARiHS framework [10], while the remaining dimensions represent a broader view of context that included additional aspects: social capital, informal interactions, formal interactions, resources, and organizational slack (staff, space, and time). Estabrooks et al. [14] reported that bivariate associations between IRU (which the ACT was developed to predict) and the majority of ACT concepts were statistically significant supporting construct validity. Adequate internal consistency was reported [14]. Individual scores from nurses were averaged to provide unit-level scores of organizational context (e.g., the nurses’ assessment of leadership on the unit) [14].

#### Research use variables

Instrumental Research Use (IRU) and Conceptual Research Use (CRU) were the “independent” predictor variables and were included in the staff survey. IRU (i.e., direct use of research) was measured using one item on a five point scale where 1 = never use and 5 = almost always use [15]. The IRU item has been shown to be acceptable to respondents [16]. CRU (i.e., indirect use of research) was measured using five items on the same scale as IRU; the mean of the five items was calculated to determine an overall CRU score. A unit level score of IRU and CRU was calculated by averaging across the scores of all nurses on the units [17].

#### Pain outcome variables

The three pain outcomes were collected from the EPIQ study and included: (a) pain assessment; operationalized as whether pain was assessed using a validated tool (Scored Yes = 1 or No = 0), (b) pain management; operationalized as whether pain was treated during a painful procedure using evidence-based pharmacologic, physical, psychological pain relieving strategies (Scored Yes = 1 or No = 0) and (c) pain intensity of a hospitalized child during a painful procedure. Pain assessment and management data were expressed as a proportion of children on each hospital unit that had pain assessed or managed. Pain intensity was assessed using validated, age-appropriate measures: the Premature Infant Pain Profile (PIPP) [18], the Faces, Legs, Arms, Cry, Consolability (FLACC) Scale [19], the Faces Pain Scale-Revised (FPS-R) [20] and the Numerical Rating Scale (NRS) [21]. These data were then expressed as the mean pain intensity for each hospital unit.

### Data collection

Pain assessment and management data in the previous 24 h were retrieved from medical records of children on each of the 32 participating units immediately following EPIQ completion by a research nurse. All pain process variables were directly entered into the Canadian Pediatric Pain Research Network database [22]. Six months post EPIQ completion, pain intensity (primary outcome) was assessed during a scheduled painful procedure by a trained pain expert using one of the four aforementioned validated pain measures. Patients were recruited consecutively based on eligibility and parental or patient consent.

#### Organizational context data

Were collected using an online version of the ACT at Baseline: Time 1 (May–August 2008) and at EPIQ Intervention Completion: Time 2 (April-August 2011). The same 32 hospital units were sampled at both time points but individuals completing the survey were not linked, thus preventing combining data or comparing respondents across time as the samples were not independent. In this paper, we report on organizational context at Time 2 as these represented the most current data. We focused on nurses only, as they were the largest respondent group, and most of the ACT validation studies to date have focused on nurses [14]. Eligible nurses were asked to complete the ACT by the research nurse at each site. The research nurse distributed survey packages that included a letter explaining the study and a card with a password and Uniform Resource Locator (URL) to access the online survey. Return of the surveys implied consent to participate. The nurse survey also included the IRU and CRU variables and demographic questions.

### Data analyses

The organizational context-dependent effects of research use (the independent variable) on the pain outcome variables were assessed using generalized estimating equations in SAS v 9.3 (Cary, NC) while controlling for the clustering of patients within units, unit type (medical, surgical, and critical care) and intervention group (EPIQ vs standard care). Because the pain assessment and pain management variables are dichotomous, the corresponding models were constructed using a binomial distribution and logit link (analogous to logistic regression), while the pain intensity variable was modeled with a normal error distribution and identity link (analogous to linear regression).

A median-split was reported for each context variable by coding units above the context-median as 1.0 and units at or below the median-context as 0.0. For each combination of dependent variable (e.g., pain assessment), context (e.g., leadership), and type of research use (e.g., IRU) three equations were estimated. The first and second equations examined the effect of research use on a pain outcome specifically for units lower than, or units higher than, the median of a context variable. Differential effectiveness of IRU (or CRU) in the low versus high contexts signal contextual moderation of the effect. The third equation used the full range of context values, but examined contextual moderation by assessing an interaction term created from a context variable and IRU (or CRU). The significance of the interaction term signals context dependence of IRU’s (or CRU’s) effect on the pain outcome.

## Results

### Influence of context on the effect of research use and pain assessment

Six of the 10 ACT variables (i.e. culture, social capital, informal interactions, structural and electronic resources, organizational slack [space and time]) significantly moderated the effect of IRU and pain assessment after accounting for the mean differences between the medical, surgical and critical-care units on pain assessment, mean differences on whether the unit received the EPIQ intervention or standard care, and the mean-similarity on nurse survey scores for nurses in the same unit (Moderation Test IRU and Valid Pain Assessment, Table 2). There was a significant difference in below-median and above-median context units for culture, social capital, informal interactions, structural and electronic resources, slack space, and slack time. These differences supported increased research use and increased pain assessment in the above-median context units.

**Table 2:** Effect of Research Utilization on Pain Assessment Stratified by Context; and Testing for Moderator Effects

Context	Instrumental Research Utilization (IV)				Conceptual Research Utilization (IV)			
	At or below the Median of the corresponding Context variable	Above the Median of the corresponding Context variable	Moderation Test (i.e. effect of IRU on Valid Pain Assessment^a^ between low and high Contexts)		At or below the Median of the corresponding Context variable	Above the Median of the corresponding Context variable	Moderation Test (i.e. effect of CRU on Valid Pain Assessment ^a^ between low and high Contexts)	
	OR (95% CI)	OR (95% CI)	*χ* ^2^	*p*-value	OR (95% CI)	OR (95% CI)	*χ* ^2^	*p*-value
Leadership	0.71 (0.33 to 1.53)	1.92 (1.08 to 3.40)	3.80	0.052	0.86 (0.42 to 1.78)	1.51 (0.81 to 2.79)	1.25	0.263
Culture	0.72 (0.33 to 1.58)	2.80 (1.52 to 5.13)	4.80	0.029	0.62 (0.22 to 1.75)	2.26 (1.27 to 4.04)	1.71	0.002
Evaluation	0.87 (0.43 to 1.75)	1.38 (0.66 to 2.89)	1.56	0.212	0.82 (0.43 to 1.58)	0.95 (0.36 to 2.52)	1.31	0.252
Social Capital	0.89 (0.48 to 1.67)	4.82 (2.32 to 10.01)	6.13	0.013	0.74 (0.40 to 1.36)	7.74 (3.02 to 19.81)	16.29	<0.001
Informal interactions	0.30 (0.14 to 0.64)	6.95 (3.12 to 15.47)	32.66	<0.001	0.52 (0.27 to 1.02)	6.42 (1.52 to 27.11)	2.58	0.108
Formal interactions	0.99 (0.48 to 2.04)	1.51 (0.78 to 2.93)	1.51	0.219	0.75 (0.36 to 1.56)	0.84 (0.37 to 1.91)	0.64	0.422
Resources	0.17 (0.08 to 0.36)	6.85 (3.40 to 13.82)	50.66	<0.001	0.40 (0.22 to 0.72)	35.10 (10.56 to 116.69)	4.25	<0.001
Slack Space	1.19 (0.61 to 2.33)	3.39 (1.75 to 6.57)	5.64	0.018	1.72 (0.96 to 3.08)	7.49 (2.68 to 20.97)	3.65	0.056
Slack Staff	1.95 (1.07 to 3.54)	1.02 (0.43 to 2.43)	1.74	0.187	1.20 (0.69 to 2.07)	12.28 (1.65 to 91.41)	2.57	0.109
Slack time	0.64 (0.30 to 1.37)	2.45 (1.30 to 4.59)	11.86	0.001	0.37 (0.18 to 0.74)	2.88 (1.30 to 6.37)	11.64	0.001

*Note: IV* Independent Variable, *DV* Dependent Variable = Pain Assessment, *χ* ^*2*^ Chi Square, *OR* Odds Ratio, *CI* Confidence interval

^a^ *N* = 964 children between low and high context units = 964 children between low and high context units

Taking into account the same mean differences and similarities as IRU, there was a significant difference in the effect of CRU and pain assessment (Moderation Test CRU and Valid Pain Assessment, Table 2). There was a significant difference in below-median work contexts and above-median work context units in four of the same ACT variables as IRU, including culture, social capital, structural and electronic resources, and slack time. Again, these differences demonstrated increased research use and increased pain assessment in the above-median context units. These differences can be detected as significant interactions, although they are challenging to interpret. Overall, using research, either directly or indirectly increased the probability of valid pain assessment when the work environments were more favorable (e.g. strong leadership, informal interactions and culture), and had essentially no effect or mixed effects in less favorable contexts.

### Influence of context on the effect of research use and pain management

No context variables moderated the effect of IRU on the pain management outcome (Moderation Test IRU and Pain Management, Table 3). IRU had no effect on pain management in either below-median or above-median contexts. After accounting for the same mean differences and similarities as the pain assessment analyses above, two ACT variables (evaluation and formal interactions) moderated the effect of CRU and pain management (Moderation Test – CRU and Pain Management, Table 3). In contrast to pain assessment, significant decreases in pain management were found when CRU increased.

**Table 3:** Effect of Research Utilization on Pain Management Stratified by Context; and Testing for Moderator Effects

Context Variables	Instrumental Research Utilization (IV)				Conceptual Research Utilization (IV)			
	At or below the Median of the corresponding Context variable	Above the Median of the corresponding Context variable	Moderation Test (i.e. effect of IRU on Pain Management^a^ between low and high Contexts)		At or below the Median of the corresponding Context variable	Above the Median of the corresponding Context variable	Moderation Test (i.e. effect of CRU on Pain Management ^a^ between low and high Contexts)	
	OR (95% CI)	OR (95% CI)	*χ* ^2^	*p*-value	OR (95% CI)	OR (95% CI)	*χ* ^2^	*p*-value
Leadership	0.93 (0.33 to 2.59)	0.39 (0.18 to 0.85)	1.43	0.231	0.21 (0.08 to 0.59)	0.30 (0.12 to 0.73)	0.09	0.762
Culture	0.64 (0.23 to 1.77)	0.63 (0.29 to 1.37)	0.01	0.972	0.08 (0.02 to 0.46)	0.39 (0.19 to 0.80)	3.07	0.080
Evaluation	0.72 (0.30 to 1.71)	0.41 (0.14 to 1.18)	0.36	0.549	0.32 (0.14 to 0.76)	0.03 (0.004 to 0.17)	5.81	0.016
Social Capital	0.44 (0.19 to 1.00)	0.68 (0.27 to 1.71)	0.69	0.407	0.27 (0.11 to 0.67)	0.27 (0.10 to 0.75)	0.01	0.929
Informal interactions	1.28 (0.44 to 3.74)	1.11 (0.42 to 2.94)	0.001	0.990	0.45 (0.18 to 1.15)	0.27 (0.04 to 1.89)	1.25	0.264
Formal interactions	0.69 (0.29 to 1.65)	0.49 (0.20 to 1.22)	0.17	0.680	0.32 (0.13 to 0.80)	0.07 (0.01 to 0.29)	4.61	0.032
Resources	0.65 (0.22 to 1.89)	0.79 (0.34 to 1.82)	0.08	0.784	0.30 (0.12 to 0.74)	0.44 (0.10 to 1.98)	0.04	0.841
Slack Space	0.60 (0.25 to 1.42)	0.54 (0.24 to 1.24)	0.040	0.842	0.32 (0.15 to 0.70)	0.09 (0.02 to 0.39)	3.82	0.051
Slack Staff	0.48 (0.23 to 1.01)	1.25 (0.37 to 4.16)	0.86	0.354	0.27 (0.13 to 0.56)	0.07 (0.004 to 1.10)	0.35	0.554
Slack time	0.65 (0.26 to 1.62)	0.64 (0.28 to 1.44)	0.02	0.885	0.22 (0.09 to 0.57)	0.25 (0.07 to 0.83)	0.01	0.904

*Note: IV* Independent Variable *DV* Dependent Variable = Pain Management, *χ* ^*2*^ Chi Square, *OR* Odds Ratio *CI* Confidence interval

^a^ *N* = 964 children between high and low context units

### Influence of context on the effect of research use and pain intensity

After accounting for the mean differences and mean-similarities noted above, 9 of 10 ACT variables (leadership, culture, evaluation, social capital, informal interactions, formal interactions, structural and electronic resources, organizational slack staff, and slack time) significantly moderated the effect of IRU and pain intensity (Moderation Test IRU and Pain Intensity, Table 4); all supported the above-median context units. Conversely, only informal interactions and organizational slack space moderated the effect of CRU and pain intensity.

**Table 4:** Effect of Research Utilization on Pain Intensity Stratified by Context; and Testing for Moderator Effects

Context Variables	Instrumental Research Utilization (IV)				Conceptual Research Utilization (IV)			
	At or below the Median of the corresponding Context variable	Above the Median of the corresponding Context variable	Moderation Test (i.e. effect of IRU on Pain Intensity^a^ (DV) between low and high Contexts)		At or below the Median of the corresponding Context variable	Above the Median of the corresponding Context variable	Moderation Test (i.e. effect of CRU on Pain Intensity^a^ between low and high Contexts)	
	β (95% CI)	β (95% CI)	*χ* ^2^	*p*-value	β (95% CI)	β (95% CI)	*χ* ^2^	*p*-value
Leadership	−0.70 (-1.87 to 0.46)	1.18 (0.22 to 2.14)	7.03	0.008	−0.26 (-1.34 to 0.83)	0.62 (-0.42 to 1.66)	1.02	0.313
Culture	−0.68 (-1.85 to 0.49)	1.28 (0.28 to 2.29)	5.81	0.016	0.23 (-1.30 to 1.76)	0.64 (-0.31 to 1.59)	0.32	0.569
Evaluation	−1.02 (-2.17 to 0.14)	1.44 (0.29 to 2.59)	6.30	0.012	−0.55 (-1.64 to 0.54)	0.37 (-1.19 to 1.93)	0.81	0.368
Social Capital	−0.45 (-1.45 to 0.55)	1.39 (0.26 to 2.53)	5.27	0.022	−0.39 (-1.36 to 0.58)	1.07 (-0.14 to 2.28)	3.54	0.060
Informal interactions	−1.01 (-2.26 to 0.24)	1.47 (0.32 to 2.62)	7.11	0.008	−0.42 (-1.56 to 0.73)	1.18 (-0.94 to 3.31)	4.63	0.032
Formal interactions	−1.12 (-2.26 to 0.03)	1.58 (0.53 to 2.62)	9.10	0.003	−0.51 (-1.67 to 0.65)	0.83 (-0.48 to 2.15)	2.14	0.143
Resources	−1.04 (-2.28 to 0.19)	1.41 (0.39 to 2.43)	9.62	0.002	−0.44 (-1.44 to 0.56)	1.45 (-0.26 to 3.17)	3.54	0.060
Slack Space	−0.48 (-1.51 to 0.56)	1.09 (0.02 to 2.16)	3.76	0.053	−0.52 (-1.42 to 0.38)	1.61 (0.01 to 3.22)	5.22	0.022
Slack Staff	−0.30 (-1.28 to 0.68)	2.07 (0.70 to 3.43)	6.91	0.009	0.26 (-0.66 to 1.17)	0.25 (-2.86 to 3.35)	0.01	0.963
Slack time	−1.32 (-2.57 to -0.07)	1.79 (0.86 to 2.72)	15.01	<0.001	−0.18 (-1.36 to 1.01)	0.84 (-0.34 to 2.03)	1.53	0.216

*Note*: *IV* Independent Variable, *DV* Dependent Variable = Pain Intensity, *χ* ^*2*^ Chi Square, *OR* Odds Ratio, *CI* Confidence interval, *χ* ^*2*^ chi-square, *β* beta-non-standardized slopes/coefficients

^a^ *N* = 640 children between low and high context units

## Discussion

### Context, research use and pain assessment and management

In this study, organizational context was an important factor in moderating the effect of nurses’ use of research and pain assessment and pain intensity in children; and less so for pain management.

#### Pain assessment

The role of organizational context in moderating the effect of IRU (and to a more limited degree CRU) and pain assessment was greater in units with higher organizational context scores. As IRU involves concrete actions, such as the generation or implementation of policies and procedures, the direct use of research aligns with best practice guidelines where nurses are expected or mandated to use validated measures to assess pain. Use of a valid pain assessment measure may represent a quality indicator of optimal nursing practice in many settings. Also, it is likely easier to change and sustain pain assessment practices as they can be more easily incorporated or routinized in care (e.g., included with the regular assessment of vital signs), where they are less influenced by individuals’ attitudes and beliefs (e.g. about the effectiveness of pain management strategies).

Organizations have placed a high value on quality improvement plans for priority outcomes such as pain. These efforts have resulted in a culture based on low or “no” tolerance for suboptimal pain assessment and management practices and formalized quality improvement plans. These initiatives are designed to strengthen leadership, engage health care professionals in interactions with patients and other health care professionals, and improve the delivery of efficient and safe care within the practice context. Franck et al. [23] have reported that factors within the practice context, as well as health care professionals’ motivation and interpersonal dynamics may influence compliance with assessing and detecting pain in clinical practice. However, the emphasis on pain assessment is based on the assumption that conducting pain assessments on a regular basis will lead to optimal pain management by healthcare professionals and thus lower pain intensity for patients. This focus and valuing of regular pain assessment may not yield the subsequent pain management behaviours that will ultimately result in improved patient outcomes. Further investigation into the role of organizational context and its effect on research use and pain outcomes may shed some light as to when and where pain assessments are completed and the relationships between pain management and clinical outcomes.

#### Pain management

There were fewer significant interactions between CRU and pain assessment and pain management outcomes and the results were variable. In the units with above-median context scores, the effect of CRU (evaluation and formal interactions) and pain management was similar to IRU. In contrast, the influence of organizational context on the effect of CRU and pain management in units with high and low context scores was not supported. CRU is less concrete than IRU and is influenced by health care professionals’ beliefs, attitudes, and opinions [16]. Even with increased knowledge gained from research use, health care professionals’ decision making on how to use this knowledge to manage pain requires planning and effort, much more so than pain assessment, which is more straightforward for both the novice and expert nurse. Furthermore, in this study, pain management data were obtained by chart review, where non-pharmacological strategies to manage procedural pain (e.g., skin-to-skin care for infants, distraction for older children) are often not well documented [5]. It is always challenging to determine whether lack of pain management evidence is due to a lapse in documentation or to intervention implementation failure. Pain management involves recollection of recent evidence, tailoring evidence on one or a combination of strategies based on the needs of the patient and family, and decision-making from a variety of options that may differ across practice settings. The selection of pain management strategies is highly dependent on availability where feasibility, cost, knowledge, and acceptability may all play a role.

### Context, research use, and pain intensity

The role of organizational context in moderating the effect of research use and the clinical outcome, pain intensity, is perplexing. In units with higher context scores, increased research use was associated with higher pain intensity scores. One would expect that greater implementation of research would be associated with more frequent and valid pain assessment that, in turn, would stimulate greater use of management interventions that would be associated with children experiencing less pain (i.e. decreased pain intensity scores). However, this was not the case in this study. In our study, higher IRU tended to reduce pain intensity. However, in units with less-favorable contexts (e.g. less leadership support, less formal and informal interactions and fewer resources), higher research use was not particularly effective in decreasing pain intensity (Tables 4 and 5). In contrast, the statistically significant and consistently positive association of IRU with increasing pain intensity in above-median contexts was unanticipated. The consistently statistically significant interaction terms reaffirm that the differences in IRU effects are likely to be real.

**Table 5:** Definitions of Context Variables Adapted from the Alberta Context Tool Developed by Estabrooks et al. [14]

Context Variable	Definition
Leadership	The actions of formal leaders in the unit that influence change and improve unit practice
Culture	The way that ‘we do things’ in the work units to reflect work culture
Evaluation	The process of using data to assess group/team performance
Social Capital	The stock of active connections among people (e.g., bonding, bridging, and linking)
Formal Interactions	Formal exchanges between individuals through scheduled activities to promote the transfer of knowledge
Informal Interactions	Informal exchanges between individuals to promote the transfer of knowledge
Structural/Electronic Resources	The structural and electronic components that facilitate the accessibility and use of knowledge
Organizational Slack Staff	The availability of staff to promote successful adaptation to meet internal and external pressures
Organizational Slack Time	The availability of time to promote successful adaptation to meet internal and external pressures
Organizational Slack Space	The availability of space, to promote successful adaptation to meet internal and external pressures

A possible explanation could be that with greater knowledge through research use and with greater attention to completing pain assessment using a validated measure, health care professionals were able to identify (and likely document) higher pain intensity (moderate to severe). However, with decreased implementation of pain management strategies (as seen with CRU), pain intensity scores would be higher. If CRU is contributing to the use of different but equally effective pain management strategies (e.g., non-pharmacologic strategies), there should be no general elevation in pain intensity with higher CRU. In future, the focus needs to move from simply identifying children with moderate to severe pain, to better understanding the complexity of pain management within a particular practice setting. Increased understanding will result in finding ways to engage health care professionals in implementing effective pain management strategies to significantly decrease pain intensity.

Overall, more elements of organizational context influenced the relationship of IRU (compared to CRU) and pain outcomes. A few (e.g., culture, social capital, structural and electronic resources, and slack time) were significant across the relationship between both IRU and CRU and pain outcomes. Squires et al. [13] reported that there were more elements of organizational context that predicted CRU (*n* = 6) versus IRU (*n* = 1) while Estabrooks et al. [15] found almost queal numbers of ACT elementat influenced IRU (*n* = 4) versus CRU (*n* = 3) Some elements of organizational context (e.g., culture, social capital, structural and electronic resources, and slack time) appear to operate in similar ways for both IRU and CRU in relation to the caregiver process or best practice outcomes (e.g. pain assessment using a valid measure). Where there is lack of consistency in results for some aspects of organizational context (e.g., informal and formal interactions, and organizational slack space), a different mechanism of contextual influence may be at play. The elements of organization context measured in the ACT may be amenable to change; thus providing promise for an intervention that enables us to shift more than individual or team behaviours and to focus interventions directly on elements of the work environment. For example, improved informal communications may provide new opportunities for engaging nurses and families in efficiently initiating better coping strategies for children undergoing painful procedures. Further empirical research needs to be undertaken to better differentiate the roles of the individual context concepts in influencing research use and clinical outcomes.

### Limitations

There are several cautions that recommend a focus on the general pattern in the study results above rather than specific claims. First, although the selection of pain outcomes was determined based on a strong footing in clinical relevance, how they were operationalized (e.g., pain assessment and management as binary outcomes) may have influenced the results. Second multiple testing of variables may have led some estimates to be significant by chance (so only highly significant estimates may warrant individual attention). In future, a more stringent test of significance could be used (e.g. *p* = .01). Third, while we conceptualized that both IRU and CRU would influence pain outcomes, there is nothing about the statistical analyses that permits us to assert causal sequencing. Alternative hypotheses such as a reciprocal effect (e.g., improved clinical outcomes impact IRU and CRU) or other factors on the causal pathway may have resulted in the observed associations.

## Conclusions

We are beginning to understand the role of organizational context in influencing process and clinical outcomes. Selecting which aspects of organizational context to focus on is important and should be done after determining a unit’s context “scores” and reflecting on where the most gain can be achieved and the degree of difficulty and the available resources for any given element. Choices should reflect the growing availability of evidence for those elements of organizational context that exert the most influence on the outcome of choice. While this body of evidence is still nascent, changing elements of organizational context is an actual possibility.

Challenges to changing the practice environment include taking into account the complexity of the change process. For example, changing unit leadership or culture and improving unit resources requires considerable effort, funding and decision making that are beyond the control of the researcher and implementation team. However, changes to formal and informal communication are more realistic and feasible. Investigation into other disciplines, such as organizational learning in business literature [24], will advance our understanding of organizational context in implementation science. Concurrently, more attention needs to be paid to the priority rating of pain within hospital units and organizations, the mapping of pain onto unit initiatives and priorities and more careful consideration of the assumptions between pain assessment and management to better address pain prevention and treatment. Only when research and practice come together in behaviour changing strategies will we see improvements in individual child health and systems level outcomes.

## Abbreviations

ACT: Alberta Context Tool
CIHR: Canadian Institutes of Health Research
CRU: conceptual research use
EPIQ: Evidence-based Practice for Improving Quality
FLACC: Faces, Legs, Arms, Cry, Consolability
FPS-R: Faces Pain Scale-Revised
IRU: instrumental research use
KT: knowledge translation
NRS: Numerical Rating Scale
PIPP: Premature Infant Pain Profile
TROPIC: Translating Research On Pain in Children
URL: Uniform Resource Locator
